# SIRT1 inhibition promotes atherosclerosis through impaired autophagy

**DOI:** 10.18632/oncotarget.17691

**Published:** 2017-05-08

**Authors:** Xiaofeng Yang, Jingyuan Wei, Yanhao He, Ting Jing, Yanxiang Li, Yunfang Xiao, Bo Wang, Weirong Wang, Jiye Zhang, Rong Lin

**Affiliations:** ^1^ Department of Pharmacology, School of Basic Medical Sciences, Xi’an Jiaotong University Health Science Center, Xi’an 710061, Shaanxi, P. R. China; ^2^ Liaoning Province Academy of Analytic Science, Shenyang 110015, Liaoning, P. R. China; ^3^ Taizhou Polytechnic College, Taizhou 225300, Jiangsu, P. R. China; ^4^ Laboratory Animal Center, Xi’an Jiaotong University, Xi’an 710061, Shaanxi, P. R. China; ^5^ School of Pharmacology, Xi’an Jiaotong University, Xi’an 710061, Shaanxi, P. R. China

**Keywords:** atherosclerosis, SIRT1, deacetylation, autophagy, Atg5

## Abstract

SIRT1, a highly conserved NAD^+^-dependent protein deacetylase, plays a pivotal role in the pathogenesis and therapy of atherosclerosis (AS). The aim of this study is to investigate the potential effects of SIRT1 on AS in ApoE^–/–^ mice and the underlying mechanisms of autophagy in an ox-LDL-stimulated human monocyte cell line, THP-1. *In vivo*, the accelerated atherosclerotic progression of mice was established by carotid collar placement; then, mice were treated for 4 weeks with a SIRT1-specific inhibitor, EX-527. The atherosclerotic lesion size of EX-527-treated mice was greatly increased compared to that of the mice in the control group. Immunostaining protocols confirmed that the inhibition of SIRT1 during plaque initiation and progression enhanced the extent of intraplaque macrophage infiltration and impaired the autophagy process. *In vitro* cultured THP-1 macrophages exposed to ox-LDL were utilized to study the link between the SIRT1 function, autophagy flux, pro-inflammatory cytokine secretion, and foam cell formation using different methods. Our data showed that ox-LDL markedly suppressed SIRT1 protein expression and the autophagy level, while it elevated the MCP-1 production and lipid uptake. Additionally, the application of the SIRT1 inhibitor EX-527 or SIRT1 siRNA further attenuated ox-LDL-induced autophagy inhibition. In conclusion, our results show that the inhibition of SIRT1 promoted atherosclerotic plaque development in ApoE^–/–^ mice by increasing the MCP-1 expression and macrophage accumulation. In particular, we demonstrate that blocking SIRT1 can exacerbate the acetylation of key autophagy machinery, the Atg5 protein, which further regulates the THP-1 macrophage-derived foam cell formation that is triggered by ox-LDL.

## INTRODUCTION

Atherosclerosis (AS), a long-term inflammatory disease characterized by atheromatous plaques in the intima of the large arteries, is an underlying cause of many cardiovascular diseases, including heart attack and stroke [1, 2]. This progressive and insidious disease is the leading cause of death and morbidity among adults worldwide [3]. Control of the progression of AS remains a major challenge because of the incomplete understanding of the involved molecular pathways. Over the past decade, an explosion in research has supported that autophagy plays a critical role in the development of AS [4, 5]. Autophagy (or “self-eating”) is an evolutionarily conserved cellular mechanism by which cytoplasmic components, such as organelles and protein aggregates, are degraded and recycled *via* the lysosomal apparatus [6]. Although the regulation and function of autophagy have been studied in the context of several contributing vascular cell types, its role in macrophage biology, in particular, has been a subject of intense interest [5]. Indeed, many recent reports have indicated that macrophage autophagy contributes to lipid metabolism, secretion of inflammatory cytokines, and atherosclerotic plaque stabilization [5, 7, 8]. However, despite the increasing evidence of in macrophage autophagy in AS, its accurate mechanisms remain to be thoroughly investigated.

SIRT1, a member of the conserved sirtuin family and a key regulator in the progression of AS, exerts protective effects by inducing autophagy, a well-known survival mechanism [9]. In 2015, Miranda *et al* found that SIRT1 provides atheroprotection in apolipoprotein E-deficient (ApoE^-/-^) mice [10]. Additionally, Takeda and coworkers demonstrated that SIRT1 inactivation induces inflammation through dysregulating autophagy in human THP-1 cells suffering from starvation [11]. In addition, it was reported that age-related pathologies, including AS, benefit from the induction of basal autophagy through augmenting sirtuin activity. Additionally, it was further validated that SIRT1 can exert multiple cellular functions by regulating autophagy through the deacetylation of several essential autophagy-related gene products, including Atg5, Atg7, and Atg8 [12]. Nevertheless, the roles of SIRT1 deacetylase in the execution and regulation of various known components of autophagy machinery during AS progression in ApoE^-/-^ mice have not been explored sufficiently.

As mentioned above, these results suggest that by regulating autophagy, SIRT1 may also be important for modulating of AS. To confirm this hypothesis, we examined the effects of SIRT1 on the autophagy level in ApoE^-/-^ mice with perivascular carotid collar placement surgery as well as the potential role of autophagy in regulating oxidized low-density lipoprotein (ox-LDL)-induced foam cell formation after EX-527 pretreatment in THP-1-derived macrophages.

## RESULTS

### Establishment of ApoE^-/-^ mouse model of AS

During the rearing period, no apparent adverse effects were observed in any groups of ApoE^-/-^ mice. There was no significant difference in the body weight of the control groups or in the EX-527-treated groups compared with the sham group (P > 0.05, data not shown). Additionally, our result demonstrated that the administration of EX-527 (10 mg/kg, intraperitoneal injection, *i.p*.) was safe in these animals.

Perivascular collar placement and high-fat diet (HFD) administration can collectively accelerate AS in ApoE^-/-^ mice [13], and the results from our present study confirmed this. An ApoE^-/-^ mouse model for AS was induced by non-occlusive silicon collar placement around the left carotid artery. At different time points after collar application (0, 2, 4, 6, and 8 wks), the development of atherosclerotic plaques was monitored in the collared carotid artery and the aortic root using hematoxylin and eosin and Oil Red O (ORO) staining methods. At the earlier two time points (2 and 4 weeks, wks) after collar placement surgery, there was no visible atherosclerotic plaque formation *in vivo* compared to the sham group (0 week, wk). In the present study, carotid atherosclerotic plaques were not observed until 6 wks after collar placement. Of note, the H&E staining of the left common carotid sections showed that the collar-induced mice (8 wks) exhibited noticeable plaque areas, which were accompanied by increased foam cell formation compared to sham-operated mice (0 wk), as evidenced by ORO staining of the sections (figure not shown).

### EX-527 promoted atherosclerosis in ApoE^-/-^ mice

**Figure 1:**
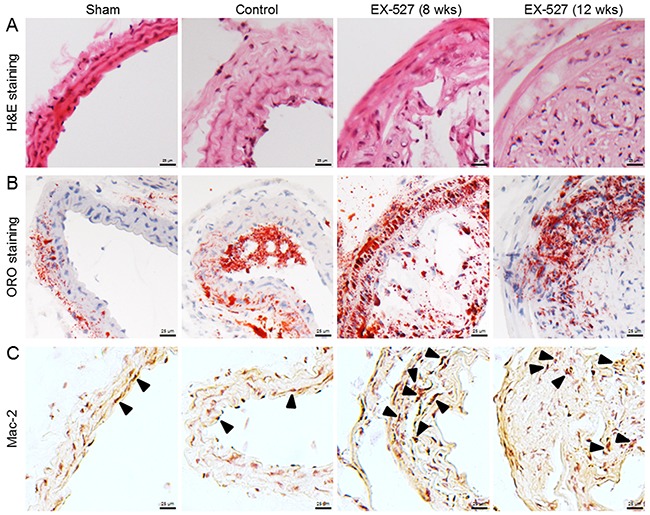
EX-527 administration promoted AS and enhanced macrophage infiltration in ApoE^-/-^ mice In both administration schedules, ApoE^-/-^ mice were treated with EX-527 at 10 mg/kg by *i.p*. for 4 wks (5 days per wk). The development of atherosclerotic plaques was monitored in the collared carotid artery using H&E **(A)** and ORO staining methods **(B)**. The effect of EX-527 on macrophage accumulation in carotid atherosclerotic plaques was detected by Mac-2 immunostaining method **(C)**. Scale bar: 25 μm. Arrows are indicative of representative Mac-2 immunostaining positively regions.

In the study of EX-527 treatment *in vivo*, to explore the effects of SIRT1 ablation on the development of atherosclerotic plaques in ApoE^-/-^ mice that underwent collar placement surgery, the dose of EX-527 (10 mg/kg) was given by *i.p*. injection to the animals for 4 wks in both protocols. H&E and ORO staining figures showed that EX-527 treated animals produced a larger plaque size and a higher percentage of foam cell formation than the control mice (8 wks, Figure 1A and 1B). Furthermore, no differences in the plaque areas or extents of foam cell formation were found between EX-527-treated mice (8 wks) and EX-527-treated mice (12 wks).

### EX-527 enhanced vascular macrophage infiltration

As plaque inflammation is of major importance for both the initiation and progression of atherosclerotic lesions, we evaluated the effects of EX-527 on plaque macrophage content using Mac-2 as a cell type-specific marker. Compared to the control group, two dose regimens of EX-527 notably increased macrophage accumulation in carotid atherosclerotic plaques (Figure 1C).

### EX-527 inhibited SIRT1 expression

**Figure 2:**
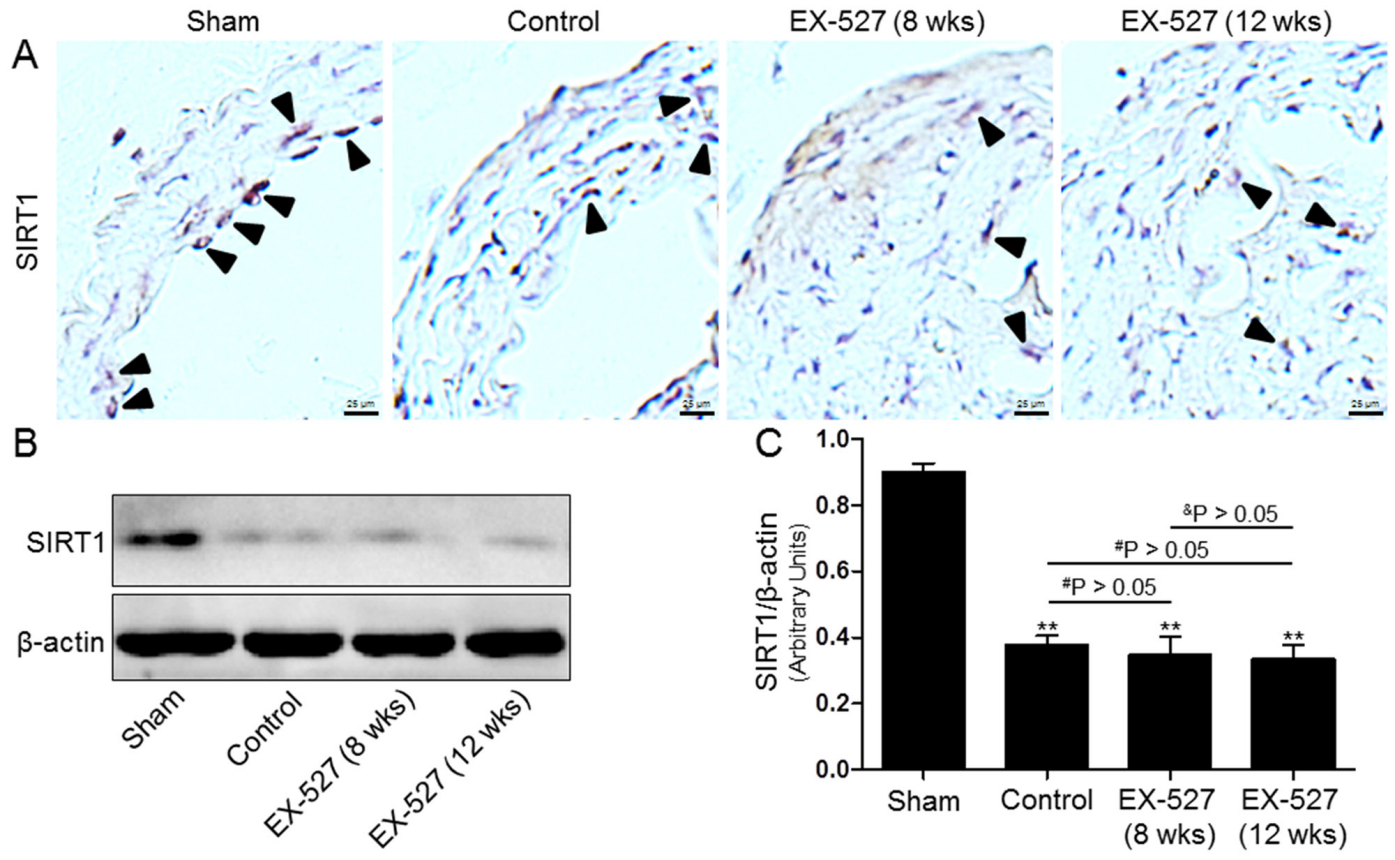
EX-527 administration inhibited SIRT1 expression in the collared carotid artery of ApoE^-/-^ mice In both treatment protocols, ApoE^-/-^ mice that underwent collar placement surgeries were treated with EX-527 at 10 mg/kg by *i.p*. for 4 wks (5 days per wk). Serial cross sections of the collared carotid arteries were immunostained with SIRT1 antibody (brown) and observed in a light microscope **(A)**. Western blot for SIRT1 protein was analyzed from the collared carotid arteries of the ApoE^-/-^ mice. β-actin was used as loading control **(B)** and **(C)**. Scale bar: 25 μm. Arrows are indicative of representative SIRT1 immunostaining positively regions. Bar graph indicates the mean ± SD (n = 3). *P < 0.05 and **P < 0.01 *vs*. Sham group; ^#^P < 0.05 and ^##^P < 0.01 *vs*. Control group; ^&^P < 0.05 and ^&&^P < 0.01 represent significant differences between EX-527 (8 wks) group and EX-527 (12 wks) group.

Next, we tested whether SIRT1 protein expression was associated with atherosclerotic lesions in an established ApoE^-/-^ mouse model of AS. SIRT1 was detected in atherosclerotic plaques in the carotid arteries using immunohistochemistry and Western blot analysis. As shown in Figure 2, our results demonstrated that SIRT1 protein expression in the control group mice was significantly reduced in the collared carotid artery compared to the sham group mice. Furthermore, after the *i.p*. injection of the SIRT1-specific inhibitor EX-527 at 10 mg/kg for 4 wks (5 days per wk), SIRT1 in the carotid plaque was measured again. However, SIRT1 protein expression of both EX-527-treated groups was not further decreased compared to that of the control group. Additionally, it was worth noting that the degree of SIRT1 expression in the plaques of pre-existing lesions of EX-527-treated ApoE^-/-^ mice (12 wks) was comparable to that of the initiating lesions of the mice receiving the same treatment at 8 wks after collar-placement.

### Autophagy level in in collared carotid artery

**Figure 3:**
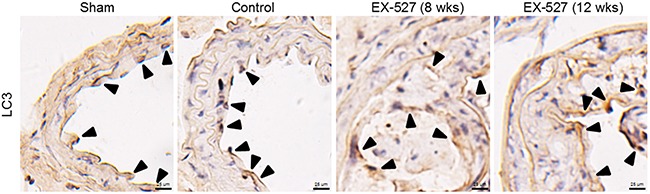
EX-527 treatment did not further change LC3 expression in the collared carotid artery of ApoE^-/-^ mice In both treatment schedules, ApoE^-/-^ mice with collar placement surgeries were treated with EX-527 at 10 mg/kg by *i.p*. for 4 wks (5 days per wk). Serial cross sections of the collared carotid arteries were immunostained with LC3 antibody (brown) and observed in a light microscope. Scale bar: 25 μm. Arrows are indicative of representative LC3 immunostaining positively regions.

We also examined the autophagy markers, including LC3-II, Beclin1, and p62 protein levels, *in vivo*. Based on the densitometry analysis of these aforementioned Western blots, there was no difference in measurements of autophagy levels between these four groups (figures not shown here). Next, we sought to verify whether autophagy changes with EX-527 treatments using an immunocytochemistry method. As shown in Figure 3, although immunostaining pictures demonstrated that LC3-positive cells were partially observed in the collared carotid artery, there were still no statistically significant differences in the LC3^+^ area/plaque ratios between these groups (P > 0.05, data not shown).

### Ox-LDL induced macrophage foam cell formation, SIRT1 inhibition, autophagy impairment, and MCP-1 production in THP-1 cells

**Figure 4:**
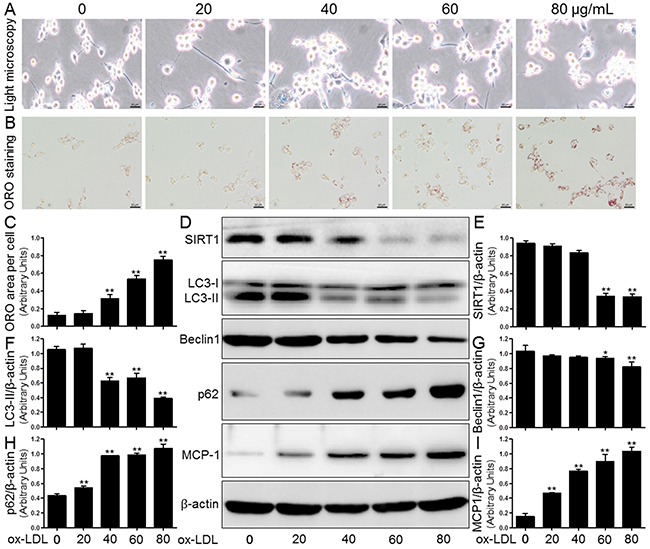
Ox-LDL induced macrophage foam cell formation, SIRT1 inhibition, autophagy impairment, and MCP-1 production in THP-1 cells Human THP-1 macrophages were exposed to 0, 20, 40, 60, and 80 μg/mL of ox-LDL for 24 hrs. Treated cells were photographed using light microscopy **(A)**. The THP-1 macrophage-derived foam cell formation was determined using ORO staining method **(B)** and **(C)**. Western blot for SIRT1, LC3, Beclin1, p62, and MCP-1 proteins were analyzed from the ox-LDL-stimulated THP-1 cells. β-actin was used as loading control **(D-I)**. Scale bar: 20 μm. Bar graph indicates the mean ± SD (n = 3). *P < 0.05 and **P < 0.01 *vs*. Cont group (0 μg/mL of ox-LDL).

First, human THP-1 cells were exposed to ox-LDL (0, 20, 40, 60, and 80 μg/mL) for 24 hours (hrs); afterwards, we confirmed the cell number and cell morphology using a light microscope. Following ox-LDL treatment, we did not observe a gradual decrease in the number of cells or any changes in the morphological characteristics (Figure 4A).

Lipid deposition in human THP-1 mononuclear cells facilitates macrophage-derived foam cell formation and ultimately accelerates the development of AS [14]. The induction of intracellular lipid accumulation by ox-LDL was determined using ORO staining. As shown in Figure 4B and 4C, the treatment of THP-1-derived macrophages with different ox-LDL concentrations (0, 20, 40, 60, and 80 μg/mL) significantly increased lipid concentrations compared to the levels in the control group (Cont). Western blot analysis showed that ox-LDL also markedly decreased the SIRT1 protein expression in a concentration-dependent manner. Both LC3 and Beclin1 crucially participate in autophagy initiation and autophagosome formation, and both serve as hallmarks of autophagy activation. We observed that ox-LDL blocked the expression of Beclin1 and LC3-II compared to control group baseline (0 μg/mL), which was again in a concentration-dependent manner. In contrast, there were gradual increases in the p62 protein expression and MCP-1 secretion in THP-1 macrophages compared to the control (Figure 4D-4I).

### Effects of SIRT1 inhibition on foam cell formation and MCP-1 expression

**Figure 5:**
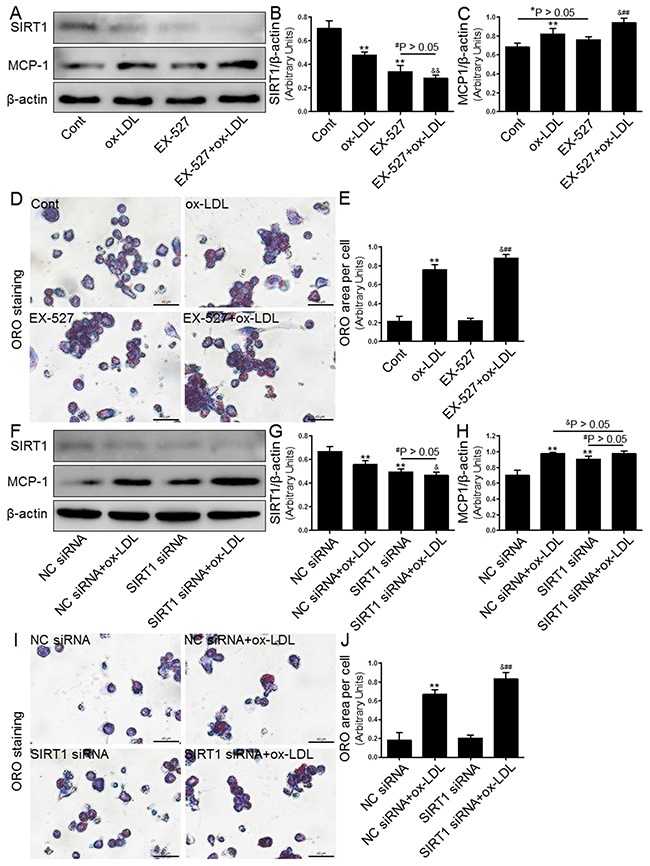
Inhibition of SIRT1 using EX-527 or SIRT1 siRNA transfection enhanced MCP-1 expression and foam cell formation Human THP-1 macrophages were pretreated with EX-527 (2 μM, for 2 hrs) or SIRT1 siRNA (20 μM, for 24 hrs), and then exposed to 80 μg/mL of ox-LDL for an additional 24 hrs. Western blot for SIRT1 and MCP-1 proteins were analyzed from the ox-LDL-stimulated THP-1 cells. β-actin was used as loading control **(A-C)** and **(F-H)**. THP-1 macrophage-derived foam cell formation was determined using ORO staining method **(D-E)** and **(I-J)**. Scale bar: 40 μm. Bar graph indicates the mean ± SD (n = 3). *P < 0.05 and **P < 0.01 *vs*. Cont group (NC siRNA group); ^#^P < 0.05 and ^##^P < 0.01 *vs*. EX-527 group (SIRT1 siRNA group); ^&^P < 0.05 and ^&&^P < 0.01 represent significant differences between ox-LDL group (NC siRNA+ox-LDL group) and EX-527+ox-LDL group (SIRT1 siRNA+ox-LDL group).

It is known that the SIRT1-mediated anti-inflammatory and lipid-modulating properties exert a protective effect in AS [15, 16]. To study the potential role of SIRT1 in lipid accumulation and MCP-1 production, THP-1 macrophages were treated with a specific SIRT1 inhibitor, EX-527 (2 μM), or transfected with SIRT1-directed small interfering RNA (siRNA, 20 μM). As shown in Figure 5, both strategies for SIRT1 inhibition, as expected, markedly reduced the SIRT1 protein expression. Because SIRT1 inactivation induces lipid metabolism disorders and inflammation, there was a significant increase in the lipid deposition and MCP-1 expression in THP-1-derived macrophages. In addition, both approaches to inhibiting SIRT1 by EX-527 or siRNA transfection yielded similar results (Figure 5A-5C and 5F-5H).

### Effects of autophagy blockage on foam cell formation and MCP-1 expression

**Figure 6:**
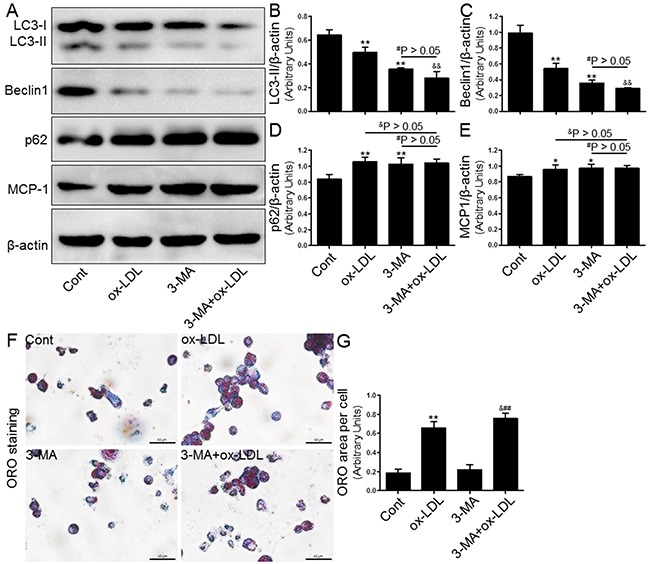
Inhibition of autophagy with 3-MA led to increased foam cell formation and MCP-1 production Human THP-1 macrophages were pretreated with 3-MA (5 μM) for 2 hrs, and then exposed to 80 μg/mL of ox-LDL for an additional 24 hrs. Western blot for LC3, Beclin1, p62, and MCP-1 proteins were analyzed from the ox-LDL-stimulated THP-1 cells. β-actin was used as loading control **(A-E)**. THP-1 macrophage-derived foam cell formation was determined using ORO staining method **(F-G)**. Scale bar: 40 μm. Bar graph indicates the mean ± SD (n = 3). *P < 0.05 and **P < 0.01 *vs*. Cont group; ^#^P < 0.05 and ^##^P < 0.01 *vs*. 3-MA group; ^&^P < 0.05 and ^&&^P < 0.01 represent significant differences between ox-LDL group and 3-MA+ox-LDL group.

**Figure 7:**
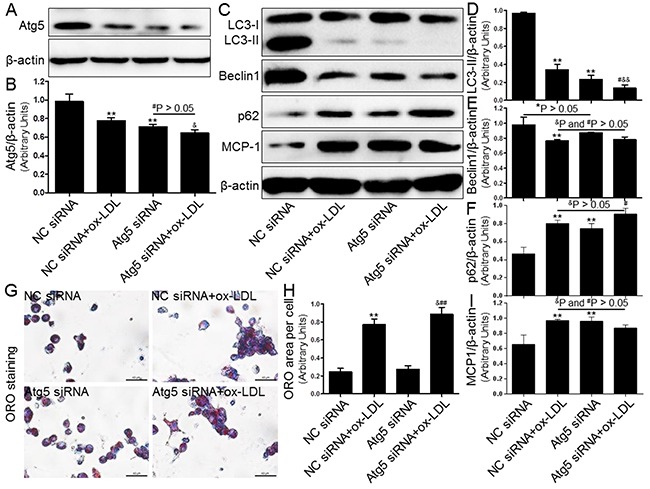
Inhibition of autophagy using Atg5 siRNA aggravated foam cell formation and MCP-1 expression Human THP-1 macrophages were pretreated with Atg5 siRNA (20 μM) for 24 hrs, and then exposed to 80 μg/mL of ox-LDL for an additional 24 hrs. Western blot forAtg5, LC3, Beclin1, p62, and MCP-1 proteins were analyzed from the ox-LDL-stimulated THP-1 cells. β-actin was used as loading control **(A-F)** and **(I)**. THP-1 macrophage-derived foam cell formation was determined using ORO staining method **(G)** and **(H)**. Scale bar: 40 μm. Bar graph indicates the mean ± SD (n = 3). *P < 0.05 and **P < 0.01 *vs*. NC siRNA group; ^#^P < 0.05 and ^##^P < 0.01 *vs*. Atg5 siRNA group; ^&^P < 0.05 and ^&&^P < 0.01 represent significant differences between NC siRNA+ox-LDL group and Atg5 siRNA+ox-LDL group.

Next, we investigated the effect of autophagy regulated by the inducer/inhibitor or gene knockdown on macrophage foam cell formation in THP-1 macrophages that were exposed to ox-LDL. In keeping with previous studies, we observed that both 3-MA (5 μM, Figure 6) and Atg5 siRNA (20 μM, Figure 7) inhibited the level of autophagy induction, which further enhanced foam cell formation. Remarkably, the effects of both 3-MA and Atg5 siRNA were of similar magnitude in independent experiments. In concert with the reduced SIRT1 expression, the inhibition of autophagy further aggravated foam cell formation (Figure 6F-6G and 7G-7H). These results indicated that autophagy can increase macrophage the uptake of ox-LDL and protect cells from lipid loading. Of note, these two interventions for regulating autophagy through the aforementioned methods did not further increase expression of MCP-1 in macrophages compared to ox-LDL group or NC siRNA+ox-LDL group according to Western blot analysis (Figure 6A and 6E; 7C and 7I).

### SIRT1 inhibition further blocked autophagy

**Figure 8:**
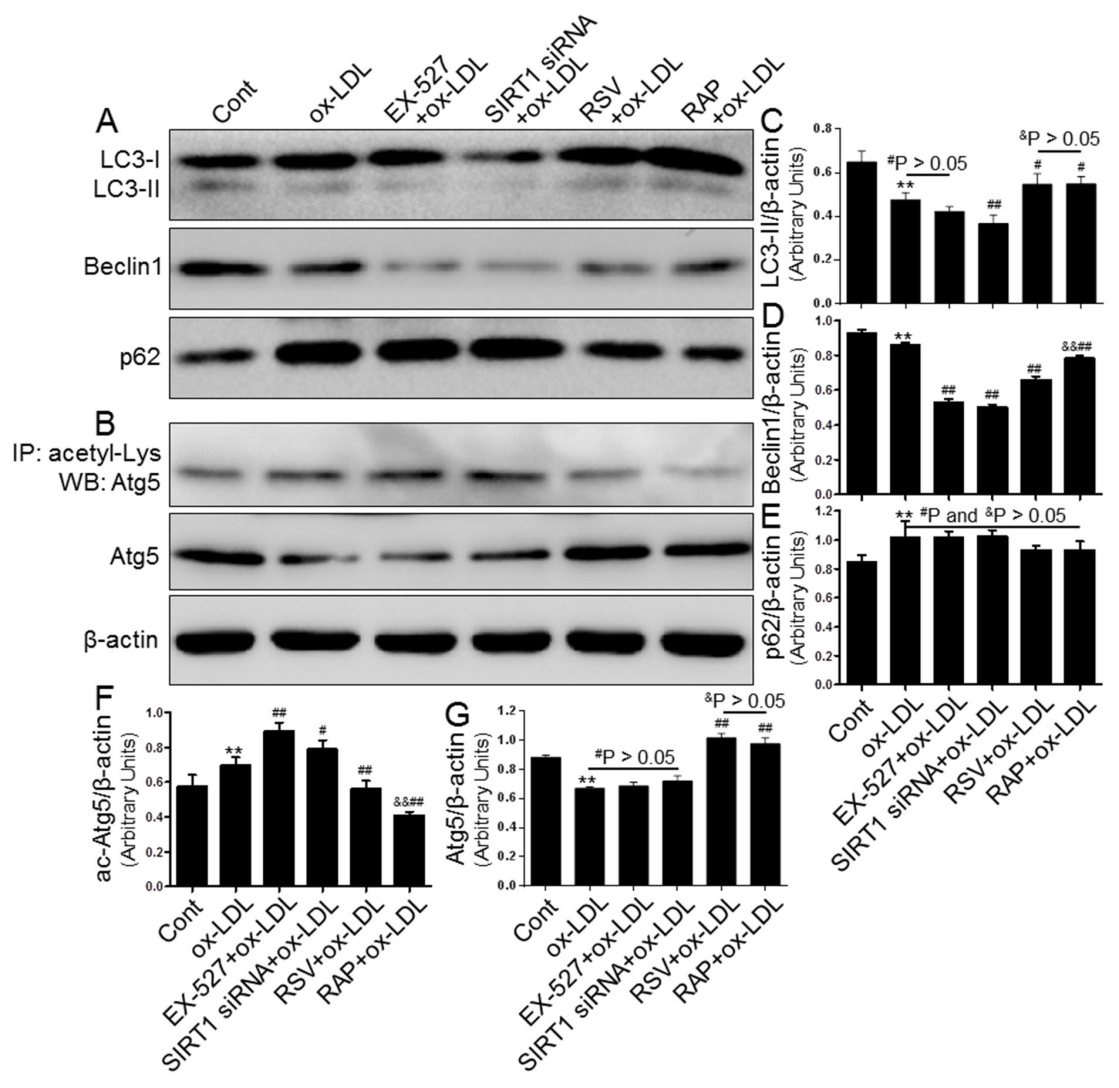
Inhibiting SIRT1 using EX-527 and SIRT1 siRNA further blocked autophagy that was down-regulated by ox-LDL exposure THP-1 macrophages were pretreated with EX-527 (2 μM, for 2 hrs), SIRT1 siRNA (20 μM, for 24 hrs), RSV (10 μM, for 2 hrs), or RAP (100 nM, for 2 hrs) and then sexposed to 80 μg/mL of ox-LDL for an additional 24 hrs. Western blot for LC3, Beclin1, p62, and Atg5 proteins and immunoprecipitation for acetyl-Lys Atg5 were analyzed from the ox-LDL-stimulated THP-1 cells. β-actin was used as loading control (**A-G**). Bar graph indicates the mean ± SD (n = 3). *P < 0.05 and **P < 0.01 *vs*. Cont group; ^#^P < 0.05 and ^##^P < 0.01 *vs*. ox-LDL group; ^&^P < 0.05 and ^&&^P < 0.01 represent significant differences between RSV+ox-LDL group and RAP+ox-LDL group.

Based on the above results, we speculated that SIRT1 inhibition would block autophagy processes, which would then stimulate MCP-1 expression and cytoplasmic lipid droplet formation. To examine this postulate, we further explored the mechanism of SIRT1-mediated autophagy suppression in THP-1 cells. First, we confirmed that both EX-527 and SIRT1 siRNA significantly inhibited autophagy induction, as evidenced by the down-regulated protein levels of LC3-II and Beclin1 as well as the decreased clearance of p62/SQSTM1 (Figure 8A). Furthermore, we observed that SIRT1 inhibition resulted in a significantly higher acetylation level of Atg5 using immunoprecipitation coupled with Western blot analysis (Figure 8B). These blots might imply that a lower autophagy level was linked with the hyperacetylation of Atg5 in the treated THP-1 cells. Simultaneously, all autophagy-related alterations and increased Atg5 acetylation were potently restored by resveratrol (RSV, a SIRT1 inducer, 10 μM) and rapamycin (RAP, an mTOR inhibitor, 100 nM). These results indicated that SIRT1 down-regulation can further aggravate autophagy impairment induced by ox-LDL, which partially occurs through facilitating Atg5 acetylation. However, the exact mechanisms by which this occurs remain to be elucidated.

### Overexpression of SIRT1 reversed ox-LDL-induced macrophage foam cell formation and autophagy impairment in THP-1 cells

**Figure 9:**
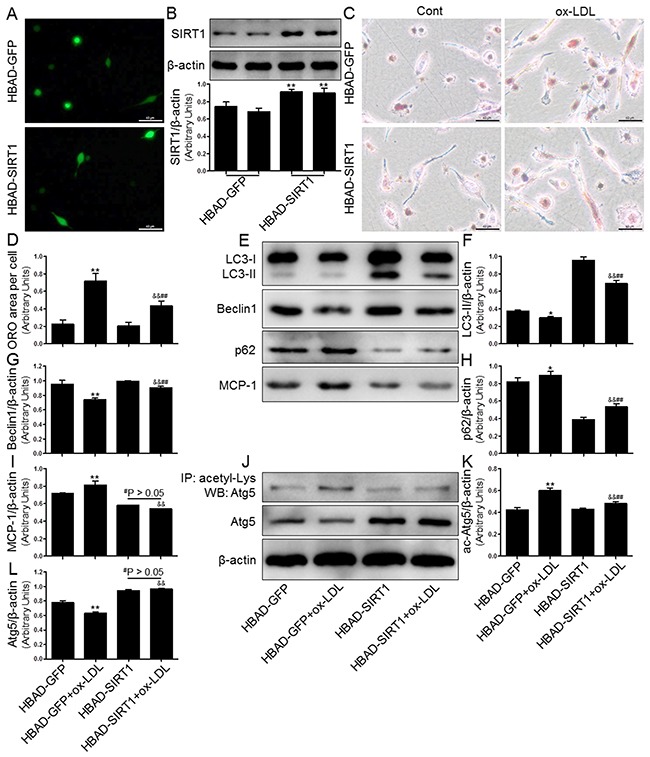
Overexpression of SIRT1 using adenoviral transfection reversed ox-LDL-induced macrophage foam cell formation and autophagy impairment in THP-1 cells Human THP-1 macrophages were transfected by SIRT1 over-expressing adenovirus (HBAD-SIRT1) or NC adenovirus (HBAD-GFP) for 24 hrs and then exposed to 80 μg/mL of ox-LDL for an additional 24 hrs. The transfected THP-1 cells were observed using an inverted fluorescence microscope **(A)** and then were harvested for transfection efficiency analysis by Western blot method **(B)**. THP-1 macrophage-derived foam cell formation was determined using ORO staining method **(C)** and **(D)**. Western blot for LC3, Beclin1, p62, and Atg5 proteins and immunoprecipitation for acetyl-Lys Atg5 were analyzed from the ox-LDL-stimulated THP-1 cells. β-actin was used as loading control **(E-L)**. Scale bar: 40 μm. Bar graph indicates the mean ± SD (n = 3). *P < 0.05 and **P < 0.01 *vs*. HBAD-GFP group; ^#^P < 0.05 and ^##^P < 0.01 *vs*. HBAD-SIRT1 group; ^&^P < 0.05 and ^&&^P < 0.01 represent significant differences between HBAD-GFP+ox-LDL group and HBAD-SIRT1+ox-LDL group.

We next investigated whether SIRT1 overexpression by adenoviral transfection also has protective effects on intracellular lipid accumulation and autophagy inhibition induced by ox-LDL challenge. To monitor and confirm the cell transfection efficiency, green fluorescent protein (GFP) and SIRT1 levels were measured using fluorescence microscopy and Western blot analysis. The results suggested that HBAD-SIRT1 promoted cellular SIRT1 expression in human THP-1 cells (Figure 9A and 9B). To determine if lipid deposition is alleviated by overexpression of SIRT1 in THP-1 cells, we observed lipid accumulation by ORO staining. As expected, HBAD-SIRT1-transfected cells inhibited macrophage foam cell formation compared to HBAD-GFP-transfected cells (Figure 9C and 9D). Moreover, Western blot analysis revealed autophagy promotion in THP-1 cells transfected with HBAD-SIRT1, which was characterized by increased LC3-II and Beclin1 protein expression and decreased p62 levels. Notably, consistent changes were observed in THP-1 cells transfected with HBAD-SIRT1 or HBAD-GFP, regardless of exposure to ox-LDL. These results suggested that HBAD-SIRT1 transfection upregulated the autophagy process in THP-1 cells (Figure 9E-9I). We further explored the SIRT1-dependent deacetylation of Atg5 in THP-1 cells overexpressing SIRT1. Our results showed that ac-Atg5 levels increased in response to HBAD-SIRT1 transfection. Additionally, in the presence of SIRT1 overexpression, ox-LDL did not further increase the degree of ac-Atg5 expression in THP-1 cells (Figure 9J-9L).

## DISCUSSION

In this study, we confirmed several novel findings for collar-induced carotid AS in ApoE^-/-^ mice and ox-LDL-stimulated foam cell formation in human THP-1 cells as follows: (1) perivascular carotid collar placement surgery accompanied by HFD administration contributes to the rapid development of AS that is associated with decreased LC3 expression and increased macrophage recruitment in atherosclerotic plaques, whereas both trends are further exacerbated by EX-527 treatment; and (2) the interaction between SIRT1 inactivation and autophagy inhibition is critical for accumulated lipid in THP-1-derived macrophages, implicating that SIRT1 contributes to Atg5 deacetylation. To the best of our knowledge, these results demonstrated that blocking SIRT1 was a negative factor for AS development in ApoE^-/-^ mice as well as an adverse regulator of lipid-droplet formation in THP-1 cells *via* disturbing autophagy induction.

Although SIRT1 deacetylase is an important regulator of AS development that has been considered for decades [17], some authors recently found direct evidence that SIRT1 exerts protective roles in AS, as well as provides an avenue for novel therapeutics. Chen *et al*. found that SRT1720, SIRT1 activator, can attenuate AS by angiotensin II induction in ApoE^-/-^ mice through inhibiting the SIRT1-mediated vascular inflammatory response [18]. In 2015, Miranda *et al*. indicated that another activator for SIRT1, SRT3025, can provide atheroprotection in ApoE^-/-^ mice [10]. Similarly, Kim *et al*. suggested that a SIRT1-dependent deacetylation process might be involved in decreasing the MMP2 release in VSMCs that are stimulated with RSV, which is also a SIRT1 activator [19]. Growing evidence has indicated that pharmacological activation of SIRT1 appears promising for AS. Although substantial data demonstrate that activating SIRT1 is an advantageous strategy for preventing AS, particularly in different animal models, it is unclear whether SIRT1 inactivation by EX-527, a SIRT1 inhibitor, accelerates AS in ApoE^-/-^ mice.

In our previous study, we found that SIRT1 blocked apoptosis through the deacetylation of FoxO1 in vascular adventitial fibroblasts [20]. Reportedly, inflammatory cytokine-stimulated apoptosis and autophagy both play crucial roles in the development of atherosclerotic lesions [21]. However, the molecular basis of autophagy in the pathogenesis of AS has not been thoroughly investigated. Herein, we further explored the underlying role for the NAD-dependent deacetylase SIRT1 in regulating autophagy as well as dissected the molecular interplay between these two crucial regulators of AS. We and others [10, 22, 23] have shown that the induction of rapid AS by perivascular carotid collar placement in combination with a HFD administration in ApoE^-/-^ mice was accompanied by increased atherosclerotic plaque macrophage content and decreased SIRT1 expression as well as disturbed autophagy compared to control mice. In this study, these results were further deteriorated by the *in vivo* administration of EX-527 to ApoE^-/-^ mice. Furthermore, ox-LDL, exhibiting various atherogenic properties including the formation of foam cells and recruitment of macrophages into arteries, plays an important role in the pathogenesis of AS and has been extensively studied [24, 25]. In this regard, studies have indicated that ox-LDL can inhibit macrophage autophagy through different mechanisms: *in vivo* and *in vitro* inhibition [23, 26]. Recent studies suggest considerable roles of SIRT1 and autophagy in the initiation and progression of AS, respectively. Of note, SIRT1 plays an essential role in regulating autophagy and autophagic flux [27], although the exact mechanism of this process is not well understood.

Towards this goal, we employed human THP-1 macrophages and treated these cells with ox-LDL to facilitate foam cell formation. Consistent with previous observations, ox-LDL, an independent risk factor of AS, reduced the SIRT1 protein level and induced the impaired autophagy process [18, 23]. It was reported that SIRT1 can increase autophagic flux through deacetylating multiple essential proteins that are involved in autophagy, including Atg5 and Atg7 as well as Atg8, under starved conditions [12, 28]. In addition, the SIRT1-mediated deacetylation of FoxO1 exerts a crucial role in promoting starvation-induced autophagy in cardiomyocytes [29]. Importantly, it was also validated that the association between the SIRT1 deacetylase and the various Atg proteins is less susceptible to nutrient fluxes [28]. In the current study, our results also showed that ox-LDL increased the protein levels of LC3-II and p62, which both serve as essential markers of autophagy. Furthermore, EX-527 treatment decreased the expression of SIRT1 and correspondingly increased the acetylation of a lysine residue of Atg5, which ultimately impaired the autophagy process in THP-1 macrophages. Remarkably, several recent *in vitro* studies demonstrated that the deacetylation of LC3 by SIRT1 allows LC3 to bind with Atg7 and other elements that are involved in autophagy [30, 31]. However, to the best of our knowledge, published studies have not reported that p62 regulates autophagy when deacetylation by SIRT1 occurs on its lysine residue. In addition, SIRT1 activation protects vascular cells from ox-LDL-induced injury by regulating AMPK/SIRT1 and eNOS/SIRT1 signaling pathways, as well as others [32–36]. Based on the aforementioned observations, it was suggested that the increased acetylation of these various autophagy proteins was associated with impairment in autophagy induction. Therefore, we further concluded that AS and a potentially wide range of other age-related pathologies may exacerbate disease manifestations by inhibiting basal autophagy through suppressing SIRT1 expression with EX-527.

However, our study limitations should be noted. First, the study was performed using *in vitro* experimental approaches to uncover the molecular mechanism involved in Atg5 deacetylation. As a more complex *in vivo* microenvironment, whether SIRT1 controls the process of autophagy in ApoE^-/-^ mice requires further validation. It has been reported that strategies for enhancing the clearance of protein aggregates are likely to offer a more fruitful atheroprotective method than reducing their formation [37]. As a result, the effect of SIRT1-mediated moderate activation of autophagy and autophagic flux during AS progression should also be determined. Of note, tissue samples for Western blotting, which were prepared from the entire common carotid artery at the site of AS, limited the accurate measurement of autophagy markers and SIRT1 protein expression in mice *in vivo*. Additionally, studies are required to explore the signaling pathways underlying this mechanism.

In summary, in our present study, both in AS and macrophages suffering from ox-LDL exposure, autophagy function is an important factor of disease progression, and this induction of autophagy appears to be an atheroprotective response that reduces the plaque burden and lipid deposition. Treatment with EX-527 further reduces SIRT1 expression and blocks autophagy through interfering with the deacetylation of an essential autophagic modulator, Atg5. Moreover, our data support that sirtuin-mediated deacetylation pathways in AS and vascular cells might be a new approach for improving autophagy function. Additionally, further studies are needed to validate the therapeutic target of SIRT1 deacetylase and to clarify the underlying molecular mechanisms.

## MATERIALS AND METHODS

### Materials

Lipofectamine™ 2000 Transfection Reagent was purchased from Invitrogen (Carlsbad, CA, USA). Oxidized low-density lipoprotein (ox-LDL) was obtained from Guangzhou Yiyuan Biotech. Co., Ltd. (Guangzhou, China). Atg5 (No.: 12994), LC3A/B (No.: 12741), Beclin-1 (No.: 3495), SQSTM1/p62 (No.: 5114), SIRT1 (No.: 8469), and β-Actin (No.: 4970) primary antibodies were purchased from Cell Signalling Technology Inc. (Danvers, MA, USA). MCP-1 (No.: NBP1-07035) primary antibody was provided by Novus Biologicals, LLC (Littleton, CO, USA). Mac-2 (No.: ab53082) primary antibody was purchased from Abcam (Cambridge, MA, USA). Pan Acetyl-Lysine (No.: A2391) primary antibody was purchased from ABclonal Biotech Co., Ltd. (College Park, MD, USA). Both protease and phosphatase inhibitors, an enhanced chemiluminescence solution, and the protein A/G magnetic beads for immunoprecipitation were purchased from Biotool (Houston, TX, USA). Standard protein markers from Bio-Rad Laboratories (Hercules, CA, USA) were used as molecular weight references. Other chemical reagents were obtained from Beyotime Biotech Co., Ltd. (Shanghai, China). All these reagents were of analytical grade, unless otherwise specified.

### Animals and experimental protocols

Eight-week-old male ApoE^-/-^ mice were provided by the Laboratory Animal Center of Xi’an Jiaotong University (Xi’an, China) and were fed a HFD (16% fat and 0.25% cholesterol) for different wks. HFD was made by the Vital River Laboratory Animal Technology Company (Beijing, China). The laboratory temperature was maintained at 24 ± 1°C, and relative humidity was controlled at approximately 60%. All mice were housed in an air-conditioned room under a 12 hrs light/dark cycle (lights on from 8:00 am to 8:00 pm). Autoclaved feed and water were allowed *ad libitum*. The mice were allowed to acclimatize for 1 wk before the onset of the experiments. The animal experiment protocol was approved by the Institutional Animal Care Committee of Xi’an Jiaotong University and carried out according to the Guidelines for Animal Experimentation of Xi’an Jiaotong University and Guide for the Care and Use of Laboratory Animals published by the US National Institutes of Health (NIH Publication number 85-23, revised 2011).

As previously described [13], ApoE^-/-^ mice received a HFD for 2 wks and then a constrictive silastic collar (0.30 mm inner diameter, 0.50 mm outer diameter, and 2 mm length; Shandong Key Laboratory of Medical Polymer Materials, Jinan, China), which was placed around the left common carotid artery near its bifurcation. Mice were sacrificed after several additional wks (0, 2, 4, 6, and 8 wks) of HFD administration, and the left common carotid artery and aortic arch were excised for pathological and molecular biological analyses.

In the second part of the *in vivo* study, 2 wks after HFD administration, 50 male ApoE^-/-^ mice underwent constrictive collar placement surgeries and were randomly divided into 2 groups (n=25 each) for evaluating the roles of SIRT1 in AS. To assess the effect of SIRT1 on lesion initiation, some mice were given EX-527 four wks after collar placement. To assess the effect of SIRT1 on lesion progression, other mice were given EX-527 eight wks after collar placement. In both treatment protocols, mice were treated with EX-527 at 10 mg/kg by *i.p*. for 4 wks (5 days per wk). The dose and route of EX-527 application used in our animal study is similar to what has been described as safe and effective in murine studies by other groups [38–40].

### Histopathology and immunohistochemistry

Both carotid artery and heart samples were fixed in 4% paraformaldehyde for 24 hrs at 4°C and washed in PBS before being incubated overnight in 30% sucrose at 4°C and then embedded in O.C.T. compound and frozen at -20°C. Serial cross sections with a 7 μm thickness were stained with hematoxylin-eosin (H&E) and ORO.

Corresponding sections were incubated with the following primary antibodies: anti-Galectin 3 (diluted 1:200), anti-SIRT1 (diluted 1:100), and anti-LC3 (diluted 1:100) from Abcam and Cell Signaling Technology Inc. Appropriate IgG conjugated with peroxidase was used as the secondary antibody (Beijing Zhongshan Jinqiao Biotechnology Co., Ltd., Beijing, China). In negative controls, incubation with the above antibodies was omitted. These sections were examined with an upright light microscope (Olympus, Tokyo, Japan) and analyzed using ImageJ software (National Institutes of Health, Bethesda, MD, USA).

### Cell culture and treatments

Human monocyte cell line THP-1 was were purchased from the Cell Bank of Chinese Academy of Sciences (Shanghai, China) and maintained in RPMI-1640 medium supplemented with 10% fetal bovine serum. Cells were cultured in a humidified incubator at 37°C in a 5% CO_2_ atmosphere. For induction of cell differentiation, THP-1 cells (1×10^6^ cells/mL) were differentiated to macrophages using serum-free medium with phorbol 12-myristate 13-acetate (PMA, 160 nmol/L; Sigma-Aldrich Shanghai Trading Co., Ltd., Shanghai, China) for 24 hrs [24]. For stimulation, cells were washed twice with PBS and reincubated with fresh serum-free medium, followed by treatment with various doses of ox-LDL (0, 20, 40, 60, and 80 μg/mL) for 24 hrs at 37°C for foam cell formation.

### ORO staining

THP-1-derived macrophages were cultured in chamber slides for 24 hrs and then incubated with ox-LDL or PBS in serum-free medium for another 24 hrs. Cells were washed with PBS three times (1 min each), and stained with ORO according to previously described methods [24]. Briefly, cells were fixed with 4% (w/v) formalin for 30 min at room temperature and then washed with PBS. Macrophages after rinsing with 60% isopropanol were stained with filtered ORO solution for 10 min at room temperature (Guangzhou Yiyuan Biotech. Co., Ltd., Guangzhou, China) and then washed with 60% isopropanol again. After the treatment, macrophages were photographed under a microscope (Nikon, Tokyo, Japan).

### RNA interference

In order to knockdown SIRT1 and Atg5 (GenePharma), THP-1-derived macrophages were transfected with 20 μM of each siRNA for 24 hrs with Lipofectamine™ 2000 reagent according to the instructions of the manufacturer. A siRNA of scrambled sequence was used as a negative control (NC, 20 μM, GenePharma). The cell lysates were typically measured at the protein level using immunoblot analysis to verify knockdown efficiency by siRNA.

### Western blotting

Western blot analysis was performed according to our previous protocol [41]. Briefly, both vascular tissue samples obtained from ApoE^-/-^ mice and THP-1-derived macrophages treated with PBS or ox-LDL were harvested, and added to Cell Lysis Buffer for Western and IP with both protease and phosphatase inhibitors following the manufacturers’ instructions. The homogenate was centrifuged at 14,000 x *g* for 15 min at 4°C. The protein concentration was measured using a BCA protein assay kit according to the manufacturer's protocol. Same amounts of total protein were loaded in each lane of sodium dodecyl sulphate-polyacrylamide gel electrophoresis (SDS-PAGE) using 12% gels and then were transferred onto a polyvinylidene difluoride (PVDF) membrane. The membranes were blocked for 4 hrs with 5 % (w/v) non-fat milk powder in Tris-buffered saline containing 0.1% (v/v) Tween-20 (TBST) at room temperature and incubated overnight with the primary antibodies at 4°C. The membranes were washed with TBST, incubated with appropriate HRP-conjugated secondary anti-mouse or -rabbit antibodies for 2 hrs at room temperature, and rewashed with TBST for 30 min (three times for 10 min each). An enhanced chemiluminescence solution was used to develop the blots for 5 min. Target proteins were detected using an enhanced chemiluminescence detection system (MiniChemi, Beijing Sage Creation Science Co., Ltd., Beijing, China). Pre-determined molecular weight standards were used as markers. Relative protein expression levels were semiquantitatively performed using ImageJ software (National Institutes of Health, Bethesda, MD, USA).

### Immunoprecipitation

For immunoprecipitation analysis of different treated cells, protein lysates (2 mg) were prepared from THP-1 cells and mixed with the anti-acetyl lysine antibody (No.: A2391, 5 μL) on an ice cold shaker overnight, which was followed by the addition of adequately resuspended protein A/G magnetic beads (60 μL, Biotool) for another 2 hrs at 4°C. Immune complexes were washed three times with wash buffer and then 30 μL of elution buffer was added. After boiling in 4 x sample buffer, samples were subjected to denaturing SDS-PAGE analysis of Atg5.

### Adenoviral vector construction

The SIRT1 over-expressing adenovirus (HBAD-SIRT1) was purchased from HanBio (Shanghai, China). The NC adenovirus (HBAD-GFP) was used as a control. THP-1 cells transfected with HBAD-SIRT1 or HBAD-GFP were harvested 24 hrs after transfection for the next experiment.

### Statistical analysis

Data are shown as mean ± standard deviation (SD), and statistical differences between groups were further evaluated by Student's t-test two group comparison or two-way ANOVA for multiple groups. Differences of P < 0.05 were considered to be statistically significant.
